# Policy Lessons From Medical Responses to the COVID-19 Crisis

**DOI:** 10.1007/s10272-021-1009-2

**Published:** 2021-12-11

**Authors:** Giovanni Dosi

**Affiliations:** Institute of Economics, Scuola Superiore Sant’Anna, Piazza Martiri della Libertà 33, 56127 Pisa I, Italy

## Abstract

This article discusses the medical/therapeutical responses to the COVID-19 pandemic and their political economy context. First, the very quick development of several vaccines highlights the richness of the basic knowledge waiting for therapeutical exploitation. Such knowledge has largely originated in public or non-profit institutions. Second, symmetrically, there is longer-term evidence that the private sector (essentially big pharma) has decreased its investment in basic research in general and has long been uninterested in vaccines in particular. Only when flooded with an enormous amount of public money did it become eager to undertake applied research, production scale-up and testing. Third, the political economy of the underlying public-private relationship reveals a profound dysfunctionality with the public being unable to determine the rates and direction of innovation, but at the same time confined to the role of payer of first and last resort, with dire consequences for both advanced, and more so developing countries. Fourth, on normative grounds, measures like ad hoc patent waivers are certainly welcome, but this will not address the fundamental challenge, involving a deep reform of the intellectual property rights regimes and their international protection.

It is useful to distinguish between the direct and indirect impact of the COVID-19 pandemic. The former includes the epidemiological effects, which are modelled in Bellomo et al. (2020). The latter concerns the effects of the institutional and policy responses to it. In turn, among such effects one may further distinguish the socio-economic impact of the measures of containment and mitigation. We discuss them with their deeply asymmetric implications among social classes and groups in Dosi et al. (2020). Finally, there are the medical/therapeutical responses, which are the focus of this article.

## Medical/therapeutical facts revealed by the pandemic and the policy responses

A few months after the identification of the COVID-19 virus, there are at least eight vaccines available (Pfizer, Moderna, AstraZeneca, Sputnik V, Johnson & Johnson, Sinopharm, Sinovac, Covaxin) and at least seven others will be available very soon (Curevac, Novavax, Convidicea, EpiVacCorona, Soberana, Abdala and Mambisa).

**Giovanni Dosi**, Sant'Anna School of Advanced Studies, Pisa, Italy.

Normally, a vaccine takes years of research, development and testing. The quick release is the result of an extremely rich body of knowledge waiting for its therapeutic application. It relates to several avenues of exploration, including around sixty potential vaccines in the pipeline as of January 2021.[1] Many of them, but not all, are broadly associated with the genetic engineering paradigm and, more specifically in our case, often associated with immunotherapies for cancer. Indeed, some of the new vaccines (Pfizer, Moderna) were obtained by imaginative re-applications of mRNA studies originally concerning cancers.

Equally striking is that such knowledge largely originated from public or non-profit institutions (Oxford University, MIT, Harvard, Gamaleya Institute, University of Mainz, public Cuban laboratories, etc.) and explored either there or in spin-offs thereof (e.g. BioNTech, Moderna).

This should not be surprising. Basic research is almost entirely supported and often also performed by the public sector in both Europe and the USA. So, for example, in the USA, all 210 new chemical entities approved by the Food and Drug Administration (FDA) in the period 2010-2016 received funding, to different degrees, from the National Institutes of Health (Cleary et al., 2018).

1 A thorough discussion is in Rawat et al. (2021).

Symmetrically, there is longer-term evidence that the private sector (essentially big pharma) decreased its investment in basic research, as witnessed by the diluted output of scientific papers cited in patent applications (Arora et al., 2018). Therefore, it is not surprising that big pharma has been found largely unprepared, at least concerning basic knowledge on vaccines. Among the new molecular entities approved by the FDA since the year 2000, less than 6% concerned antibiotics or anti-viral drugs (Walker, 2020).

Attention to vaccines has always been low. Even the public-private initiative regarding AIDS vaccines, which had raised many hopes (Chataway et al., 2007), failed. Vaccines for AIDS, and later Ebola were never developed. After all, they affected "special groups" or poor populations. It is more rewarding to invest in cures which ideally make chronic otherwise acute diseases (docet the anti-retroviral drugs for AIDS). But, of course, the business is different for a virus which is quite egalitarian in terms of national per capita incomes and social classes (of the infected, not of the casualties).

In this case, the whole private sector has immediately been eager to undertake focused applied research, production scale-up and population testing in exchange for an enormous amount of financial transfers.[2] Approximate estimates suggest €8 billion from the European Union and around $18 billion in the United States. Nobody knows exactly for what: Research? Manufacturing? Testing? Advance payment of the vaccines themselves?[3]

Be that as it may, the developed Western societies ended up with a limited vaccine supply – with the exception of the USA and Israel. And the developing world – including India, which, incidentally, produces around 40% of the world vaccine supply – fared far worse.

The political economy of the public-private relationship revealed by the policy responses to the pandemic generally highlights how governments and regional institutions are often (voluntarily?) held hostage by big pharma. The few countries not rationed have been those giving in to any demands – even at the expense of others, while the EU is in a losing position despite signing pathetic contracts.

Here, we are well beyond the "regulatory capture": It is the reversal of the relationship between the state and private actors, enshrined even in the most pro-market constitutions. In this respect, however, there is a major difference between the European Union (and its member states) and the United States. The EU epitomises the complete abdication of public authorities from their functions (basically telling private actors to do whatever they deem appropriate, in exchange for whatever they ask).

Conversely, the United States has kept a thorough system of command and control in place within the framework of the Defense Production Act of 1950 (Pub.L. 81–774) that authorises the president to order the production and distribution of goods and equipment and to requisition properties deemed necessary for national security. Written large, the Act has been repeatedly invoked in reference to COVID-19 by both Presidents Trump and Biden. Failure to comply with it is a federal felony.

More specifically, regarding pandemics and other health-related threats, the Pandemic and All-Hazards Preparedness and Advancing Innovation Act (PAHPAI, Pub.L. 116-22) of 2019, which expands the Pandemic and All-Hazards Preparedness Act (PAHPA, Pub.L. 109-417) of 2006, establishes a system of responses whose philosophy is a comprehensive mix of compulsory previsions and allocation of resources to the private sector in order to comply. One of the main instruments is the Biomedical Advanced Research and Development Authority, established in 2006 under the PAHPA, which has been the main vehicle for the transfer of the roughly $18 billion mentioned above.

Essentially, the USA represents a model where the fighting of wars is privatised – which is bad enough – but the state maintains the authority to set objectives and strategies. The EU's philosophy is basically the equivalent of allocating money to recruit others to fight whatever war they themselves decide, and without even the compulsory task of winning it. (Because the market knows better.)

Developing countries are, by and large, in a much weaker situation, often plagued by incompetent and corrupt bureaucracy. Only a few have the manufacturing capabilities to make vaccines under license, and even fewer feel the political power to invoke articles 27, 31 and 73 of the Agreement on Trade-Related Aspects of Intellectual Property Rights (TRIPS) permitting exceptions to intellectual property rights (IPR) sales with compulsory licenses in the case of health and security crises.

Last, but not least, the COVID-19 pandemic crisis has dramatically highlighted the damages of the neglect or, in some countries, the retreat by the state from a universal public good – health, and the corresponding extension of the market domain (Nelson, 2005). The scenes of seriously ill patients unable to reach hospitals are unfortunately common in developing countries, but the pandemic has shown the policy-induced scarcity of public services also in developed ones.

2 Note that this represents a major discontinuity vis-à-vis the historical record of anti-flu vaccines usually developed under a regime of open science.

3 Incidentally, notice that also some patents crucial for mRNA techniques have a public origin and are detained by public institutions (e.g. University of Texas and the National Institute of Allergy and Infectious Diseases of the National Institutes of Health).

### General lessons

This pandemic will not be the last one. It is a profound sign of the changes in the relationship between humans and nature that occurred after the Industrial Revolution and rapidly accelerated over the last half century. Some scholars go as far as to say that we have entered the Anthropocene (Coriat, 2020; Crutzen, 2006).

Certainly the destruction of biodiversity, the elimination of any distance between wild and human habitats, the exponential increase in the industrial farming of animals, e.g. poultry, are all recipes for culture of viruses and bacteria mutations and their quick transmission to humans.

Even if vaccines are an ex-post mitigation and not a long-term answer, advanced societies, let alone developing ones, turned out to be largely unprepared. The fundamental reason is the deeply dysfunctional relationship between the private and the public in the generation and exploitation of innovative knowledge, in our case of health-related knowledge. And, in turn, the dysfunctionality rests upon the extent, depth and distribution of IPR.

The Bayh-Dole Act (1980) in the USA, and imitations in other countries, including the EU, which allowed for the patentability of the outcome of publicly funded research, tends to distort the search efforts of e.g. universities, which should be mainly curiosity driven. (Fortunately, the evidence supports that, at least in top universities, such distortions have not been too deep, but the risk is always there.) Public institutions generate promising "basic" knowledge that is then sold often at ridiculous prices to big pharma or incorporated into spin-offs that might generate enormous rents to successful academics. Additionally, testing is done in vitro and finally on humans. Note here the potential conflict of interest involved in a process whereby drug companies test their own products.[4] At the end, it is the public who continue to support fundamental research, while it is ultimately big pharma that masters the rates and directions of innovative activities. Finally, drugs and vaccines are sold back, directly or indirectly, to the public at prices that have little to do with either the private costs of research or the costs of production.

It is often said that the fight against the pandemic is a war. If it is, wars are too serious a business to be left to the markets. During WWII, the USA had become, for very good reasons, a nearly fully centrally planned economy. Roughly three months after Pearl Harbor, it was capable of producing circa one tank per hour (Gross and Sampat, 2020, 2021; Best and Bradley, 2020). Conversely, after the COVID-19 outbreak, California received, with delays, a largely insufficient number of faulty testing kits; after three months the Italian government was unable even to map who was able to produce masks; ventilators had been scarce for months.

### Policy lessons

The most fundamental policy lessons are long-term. The illusion of control over nature, and the use of nature as a sink (Brock and Taylor, 2005) must be reversed before it is too late.

Equally important, health must become a universal human right, and knowledge concerning health a global common good.

The crisis has shown the deep pitfalls of a health system partly or nearly fully left to the market. If health is a universal right, this must be taken care of by the public as much as, e.g. justice or public security. On the contrary, even when there is universal health coverage, like in most European countries, public hospitals have often been the prime victim of austerity policies. This must be urgently reversed. What is needed is a massive increase of the overall public expenditure for the health system and the strengthening of local hospitals and laboratories: a capillary hospital system is able to cope with widespread diseases. Basic health-related research is part of a global health mission, thus not subject to the mean calculation of cost-benefit analysis by economists.

During crises like the current one, it should be obvious that vaccines must be made available to the entire world. This, in turn, demands generalised compulsory licensing.

More fundamentally, in the near future, it is crucial to reform the prevailing system of protection of IPR and its international projection via the TRIPS agreements within the World Trade Organization. As we argue at greater length in Dosi and Stiglitz (2014), it is bad for science in developed countries, for global science, and for the economies of both developed and developing countries alike. It has been designed not to maximise innovation but to maximise rents for those who have had the good fortune of receiving a patent (and the two are not the same).

While it is not clear that IPR in general promotes innovation, there is good evidence that there may be adverse effects, especially with poorly designed tight IPR regimes: Access to life-saving medicines may be restricted as well as access to knowledge that is necessary for successful development, and even for follow-up innovation. As governments have to spend more money to purchase the drugs they need, because of reduced availability of low-cost generic medicines, other expen-

4 Typically this is also done for the majority of drugs based on low number of treated and placebo subjects, on the grounds of very weak statistical tests. Vaccines are an exception, and in the case of COVID-19 testing has become intermixed with a sort of pre-sale marketing.

ditures – from those necessary to promote growth to those devoted to alleviating poverty – are reduced. Conversely, there may be perverse links between IPR protection and income distribution.

In some circumstances, such as in the pharmaceutical industry, the evidence is particularly striking. Before TRIPS, generics obtained under loose IPR regimes were able to dramatically reduce the cost of drugs available to developing countries. A vivid illustration concerns antiretroviral drugs against HIV infections where generics were able to reduce the cost by between 70% and 98% (Coriat et al., 2006; So et al., 2014).

Especially in the case of pharmaceuticals, where patents are indeed a major mechanism of rent appropriation, I propose that the public, which, to repeat, finances and performs most of the phase I of research, ought to move all the way to phase III (i.e. experimentation on humans), and when successful, transfer to big pharma, on nonexclusive base, the license to produce – which at that point should yield costs and thus prices not be too different from marginal costs.[5] There would be three major gains.

First, the public would regain the control over the search priorities, that is on the rates and directions of innovative activities. Second, it would certainly be a reform at massive negative costs for the collectivity. Third, it would be a major equaliser in the access to lifesaving drugs between developed and developing countries.

5 President Biden's proposal to waive patents related to COVID-19 vaccines is certainly not the solution to the general problem of IPR in pharmaceuticals, but can be a significant step in the right direction. Many of the reactions are disarming. A few commentators argue, first, that the waiver would be ineffective for most developing countries because they do not possess the tacit knowledge to produce vaccines even in absence of patents, and, second, that the waiver would be a bad precedent decreasing the incentives for big pharma to do research. Of course, both points cannot apply together. (Ugo Pagano has repeatedly pointed it out in personal communication). In the substance, the first point is certainly true, but this just reinforces the argument in favour of the development of local technological capabilities – Cuban style. The second is strikingly false, in general (see above, and the discussions in Cimoli et al., 2014; Angell, 2004; Dosi and Stiglitz, 2014), and with reference to vaccines in particular. The pharmaceutical industry has a historic record of negligible interest in vaccines and it will turn to this neglect, unless flooded by public resources, in terms of both knowledge and money. For sure, proposals like Biden's trigger the "generosity" of big pharma, offering billions of doses at lower prices. Personally, I am all in favour of universal rights rather than premodern charity.
